# Quasi-one-dimensional density of states in a single quantum ring

**DOI:** 10.1038/srep40026

**Published:** 2017-01-05

**Authors:** Heedae Kim, Woojin Lee, Seongho Park, Kwangseuk Kyhm, Koochul Je, Robert A. Taylor, Gilles Nogues, Le Si Dang, Jin Dong Song

**Affiliations:** 1 Department of Opto-Mechatronics Engineering and Cogno-Mechatronics Engineering, Physics Education, RCDAMP, Pusan Nat’l University, Busan 609-735, Republic of Korea; 2 Clarendon Laboratory, Department of Physics, University of Oxford, Oxford, OX1 3PU, U.K; 3 Department of Physics, College of Liberal Arts and Sciences, Anyang University, Gyeonggi-do, 430-714, Republic of Korea; 4 Department of NANOscience, Institut Néel, CNRS, rue des Martyrs 38054, Grenoble, France; 5 Center for Opto-Electronics Convergence Systems, KIST, Seoul, 136-791, Republic of Korea

## Abstract

Generally confinement size is considered to determine the dimensionality of nanostructures. While the exciton Bohr radius is used as a criterion to define either weak or strong confinement in optical experiments, the binding energy of confined excitons is difficult to measure experimentally. One alternative is to use the temperature dependence of the radiative recombination time, which has been employed previously in quantum wells and quantum wires. A one-dimensional loop structure is often assumed to model quantum rings, but this approximation ceases to be valid when the rim width becomes comparable to the ring radius. We have evaluated the density of states in a single quantum ring by measuring the temperature dependence of the radiative recombination of excitons, where the photoluminescence decay time as a function of temperature was calibrated by using the low temperature integrated intensity and linewidth. We conclude that the quasi-continuous finely-spaced levels arising from the rotation energy give rise to a quasi-one-dimensional density of states, as long as the confined exciton is allowed to rotate around the opening of the anisotropic ring structure, which has a finite rim width.

Recently, great progress in droplet epitaxy techniques have enabled the growth of novel nanostructures^1^,^2^,^3^,^4^,^5^. In particular, semiconductor quantum rings (QRs) are of great interest as candidates for the optical Aharonov-Bohm (AB) effect^6^,^7^,^8^,^9^,^10^. As the orbital angular momentum of an electron rotating around a QR is quantized, finely-spaced energy levels appear in between the radially confined energy levels for a finite rim width. As the magnetic flux threading a QR cross section is increased, the ring orbital angular momentum of the lowest energy level as a function of external magnetic field (*B*) changes periodically. Therefore, oscillations in the ground state exciton emission energy with *B* can be observed, where the oscillation period is determined by the orbit size, rim width, and the effective mass of the electron and hole.

The morphology of QRs was observed to be different from that of an ideal ring structure, where the lateral shape ellipticity and rim height anisotropy are ignored. When a volcano-like rim anisotropy is present^11^,^12^,^13^,^14^,^15^, the wavefunction becomes localized with a crescent-like shape^16^,^17^,^18^. Provided that the degree of localization is moderate, rotational motion of an exciton around the whole circumference can be possible through tunneling. However, the condition depends on the ring morphology. As the cylindrical symmetry breaks down in the lateral structure, the orbital angular momentum no longer represents the eigenstates. In this case, the finely-spaced levels can be described by a linear combination of the quantized orbital angular momentum states, with a consequent significant modification of the selection rules. Therefore, the finely-spaced levels of a volcano-like anisotropic ring structure are different from those in a simplified ring model^4^,^19^.

According to the exciton AB oscillation of a single QR^20^, the level spacing and linewidth of the finely-spaced levels (~0.1 meV) were found to be comparable in a QR. In this case, a density of the these states (*D(ε*) ~ *ε*^*β*^) can be defined as a function of energy (*ε*). The exponent (*β*) can be estimated experimentally by considering the ratio of the radiative decay rate near the ground state with respect to the stochastic fraction of excitons contributing to recombination *r(T*), defined later. This results in a temperature dependence for the radiative recombination time (*τ*_r_ ~ *T*^*α*^). This experimental method has been used in bulk semiconductors (*β* = 0.5), quantum wells (*β* = 0), and quantum wires (*β* = −0.5), giving rise to *α* = 1.5, 1, and 0.5, respectively^21^,^22^,^23^. In this work, we have investigated *β* for a real QR in the absence of *B* in terms of *τ*_r_(*T*) and *r(T*), which were obtained by using the temperature dependence of the linewidth, the time-resolved, and time-integrated photoluminescence (PL) intensities.

## Results and Discussion

**Figure 1:**
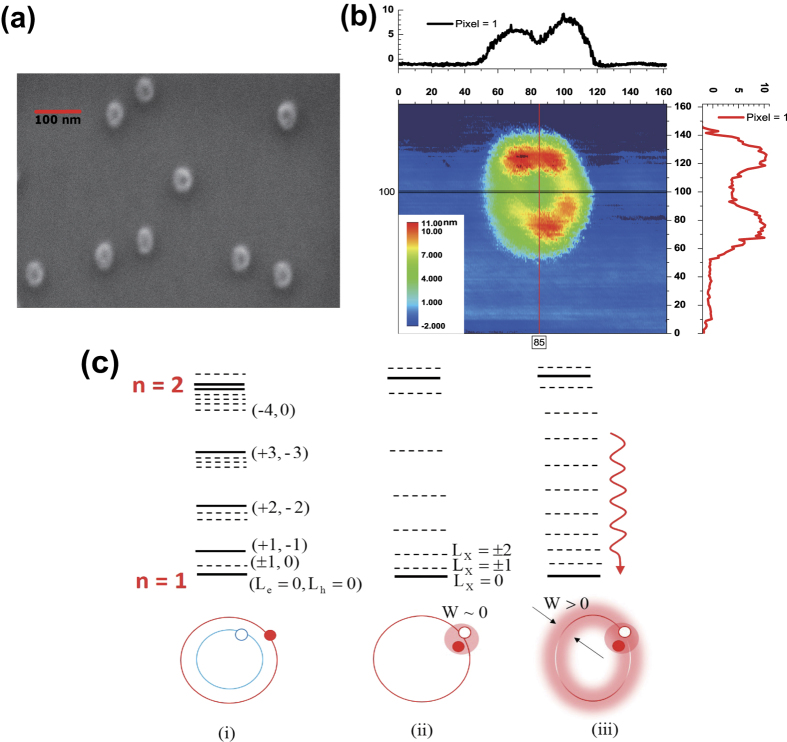
Quantum ring structures observed by FESEM (**a**) an AFM image of a single QR shows rim height anisotropy and lateral shape asymmetry (**b**). (**c**) Energy levels of an electron-hole pair in a QR are shown schematically. (i) When both the ring width (W) and the Coulomb interaction are ignored, the various levels of an independent electron and a hole are specified by two angular momenta (*L*_e_, *L*_h_) of the one-dimensional orbits (*R*_e_, *R*_h_). (ii) When the Coulomb interaction is considered, the single particle states of the center-of-mass exciton are described by the total angular momentum *L*_X_ for the widthless orbit (*R*_X_). (iii) The finely-spaced levels become modified in the presence of a finite ring width (*W* > 0), rim height anisotropy, and lateral shape asymmetry.

As shown in Fig. 1(a), ring structures were observed in a field emission scanning electron microscope (FESEM) image of an uncapped sample. However, the atomic force microscopy (AFM) image of a single QR with ~25 nm radius and ~10 nm height (Fig. 1(b)) shows that the real morphology of a ring structure is volcano-like, where the cylindrical symmetry is broken in the lateral direction and the rim height is anisotropic. On the other hand, ideal ring models are often assumed in order to explain theoretically the finely-spaced energy levels of a QR. For an electron rotating along a one-dimensional widthless orbit, the electron energy for the quantum number of ring orbital angular momentum (*L*_*e*_ = 0, ±1, ±2, …) is given by,


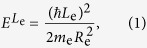


where *R*_e_, and *m*_e_ are the orbital radius and effective mass of an electron, respectively. When the Coulomb interaction between an electron and a hole is ignored, the exciton energy of a one-dimensional ring can be given by simply adding the hole energy term to equation (1) as


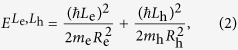


where *R*_h_ and *m*_h_ are the orbital radius and effective mass of a hole in a QR, respectively. Because the effective mass and deformation potential of an electrons and holes differ, it is likely that *R*_e_ ≠ *R*_h_, whereby the magnetic flux difference between an electron and a hole can give rise to an excitonic AB effect. In this case, the finely-spaced exciton states become optically active only when *L*_e_ + *L*_h_ = 0 (solid lines for the energy levels shown in Fig. 1(c–i)), otherwise all cases of *L*_e_ + *L*_h_ ≠ 0 are dark states (dotted lines).

When Coulomb interactions are considered, the total energy of an exciton in a QR is dominated by the center-of-mass term, giving an energy for the Coulomb-bound electron-hole pair in a QR as


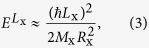


where *M*_x_ = *m*_e_ + *m*_h_, *L*_x_ = *L*_e_ + *L*_h_, and *R*_x_ = (*m*_e_*R*_e_ + *m*_h_*R*_h_)/(*m*_e_ + *m*_h_) are the total mass, ring orbital angular momentum, and orbital radius for the center-of-mass exciton, respectively. When the center-of-mass exciton rotates in a QR, all the finely-spaced levels are dark states except for the ground state *L*_x_ = 0. Provided that the level spacing is fine enough to define a density of the states for the energy, equation (3) is analogous to that of a one-dimensional confinement system, i.e., *D(ε*) ~ *ε*^−0.5^ as the level spacing increases. However, as the rim width becomes significant compared to the orbit size, the increase of the level spacing is suppressed gradually. For example, when the radial confinement potential for a finite rim is quadratic *V(r*) ~ (*r* − *R*_x_)^2^, the finely-spaced levels are equally spaced. Therefore, density of the states becomes constant with energy, *D(ε*) ~ *ε*^0^, as is the case for two-dimensional confinement.

The real confinement potential determined by QR morphology differs, however, from the quadratic form *V(r*) ~ (*r* − *R*_x_)^2^. Recently, cross-sectional scanning tunneling microscopy (X-STM) has revealed that the QR structure is anisotropic and singly-connected^11^,^12^. Based on the comprehensive structural analysis, the real QR morphology was modelled with a singly-connected volcano structure, i.e., a dip is present in the center area rather than opening and the height is anisotropic^13^,^14^,^15^. It is noticeable that the Aharonov-Bohm effect has been observed in spite of the anisotropic height and singly-connected QR structure^7^,^20^. Because the electron wavefunction decays rapidly toward the center in the singly-connected volcano-like QR, it becomes effectively identical to the wavefunction in a doubly-connected structure. Furthermore, as the rim height anisotropy breaks the cylindrical symmetry, the radial wavefunction can not be separated from the azimuthal one. In this case, the finely-spaced levels of a volcano-like anisotropic lateral confinement potential *V(r, θ*) are not the eigenstates of *L*_x_ as shown in Fig. 1(c–iii). Consequently, it is plausible to conjecture that the power factor for a real QR follows a quasi-one dimensional density of states, i.e., −0.5 < *β* < 0.

**Figure 2:**
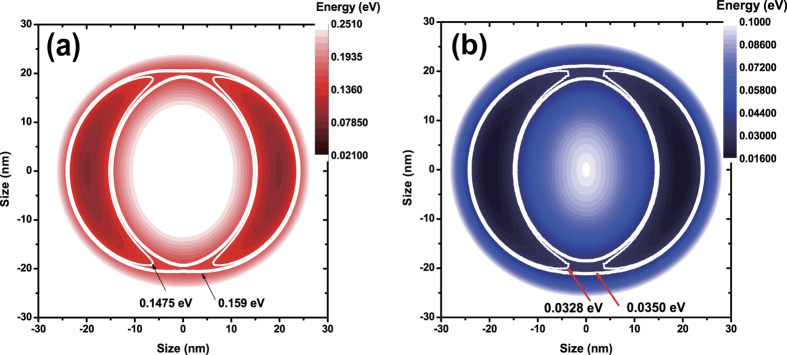
Adiabatic potential energy of a volcano-like QR for an electron (**a**) and a hole (**b**).

**Figure 3:**
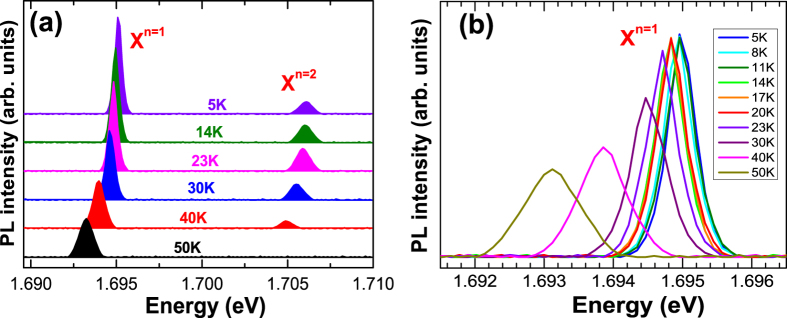
(**a**) Temperature dependence of the PL spectrum for the ground (X^*n*=1^) and first excited (X^*n*=2^) exciton states in a single QR with strong excitation (2.0 kWcm^−2^). (**b**) Temperature dependent PL spectrum for the ground exciton states (X^*n*=1^) with weak excitation (0.6 kWcm^−2^) resulting in no X^*n*=2^ PL.

Although the lateral size inhomogeneity of ensemble QDs (Fig. 1(a)) is not significant, the spectral inhomogeneity of ensemble QRs is quite broad due to the anisotropic volcano-like morphology, where the energy ranges from the bandgap of bulk GaAs up to that of AlGaAs. In this case, it was known that the wavefunction is likely to be localized in a crescent-like shape, but the degree of localization depends on the QR morphology^17^,^20^. The emission near the barrier originates from a strongly localized state in the QR. However, the crescent-like wavefunction can also extend over the whole rim of a QR with the assistance of tunneling, when the degree of localization becomes moderate. Therefore, it is important that a micro-PL spectrum is selected which is characteristic of an extended wavefunction over the whole rim. In order to evaluate the micro-PL spectrum of a single QR near ~1.695 eV, the potential energy of the electron and the hole were calculated using the volcano-like QR model (Supplementary). Provided that lateral dimensions of a QR are relatively large compared to the vertical, an anisotropic confinement potential *V(r, θ*) can be obtained by using the adiabatic approximation. This adiabatic potential for electrons and holes 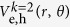 can be determined for the excited vertical quantum state (*k* = 2) (Fig. 2(a) and (b)). Anisotropy makes the confinement levels for *θ* = 0° and 90° different, as shown by white solid lines in Fig. 2(a) and (b). Although a subtle difference is present between the model and a real QR, the micro-PL spectrum of the ground exciton state (X^*n*=1^) near ~1.695 eV can be predicted, i.e., an energy difference between the PL spectrum and the bulk GaAs band gap (~176 meV) results from the total confinement energy (~184 meV) and the Coulomb interaction (~8 meV)^20^. Consequently, the PL spectrum near ~1.695 eV (Fig. 3) can be attributed to an extended wavefunction over the whole rim although its distribution is anisotropic. The subtle difference can be revised by regarding the Coulomb interaction in the presence of the anisotropy^15^.

When excitation is strong, we found that an additional high-energy PL peak becomes significant at ~10 meV above the main low-energy PL peak (Fig. 3(a)). We have confirmed that the high-energy PL peak is an excited exciton state (X^*n*=2^) by using excitation intensity dependent and time-resolved PL (Supplementary). X^*n*=2^ becomes also suppressed as the temperature increases. We have already shown^17^ that the *p* − orbital asymmetry of X^*n*=2^ gives rise to a large polarization asymmetry and a broad linewidth as a consequence of the strong coupling with phonon bath compared to X^*n*=1^. Intermediate spectra between X^*n*=1^ and X^*n*=2^, where the finely-spaced levels of a ring structure with sub-meV spacing might be seen, are extremely weak. The intra-relaxation in these levels is therefore predicted to be very fast (~10^0^ ps). This implies that the fractional radiative recombination rate with respect to thermal excitation *r(T*) within the ground state PL spectrum of X^*n*=1^ can be used to estimate the density of the states.

**Figure 4:**
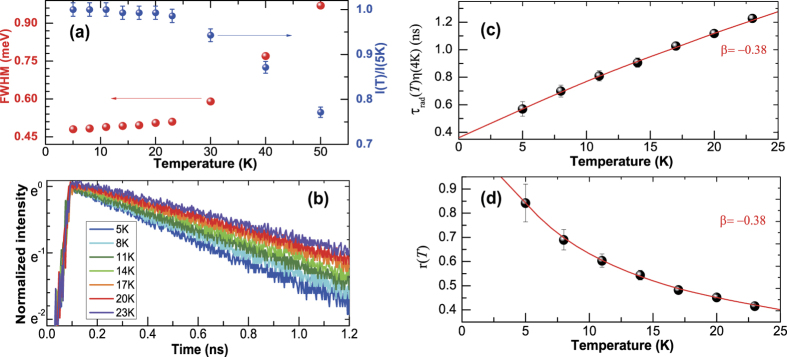
(**a**) Linewidth and time-integrated intensity of the PL spectra are plotted as a function of temperature (**b**) Time-resolved PL of a single QR measured at various temperatures, where the temperature dependence of the radiative decay time *τ*_rad_(*T*) (**c**) and *r(T*) (**d**) were obtained by calibration using the PL intensity and linewidth, respectively. The fitting parameter *β* = −0.38 suggests that in our QR we have a quasi-one-dimensional density of states.

For an optical transition between the confined ground states of an electron and a hole in a one-dimensional (1D) or two-dimensional (2D) system, the transition strength of the exciton at *K* = 0 (*F*_x_) can be determined by the oscillator strength *f*_x_ and the wavefunction of the electron with respect to the hole *ϕ*(**r**_e_ − **r**_h_) as *F*_x_ ~ *f*_x_|*ϕ*(0)|^2^. However, as the excitons are scattered by acoustic phonons, a finite linewidth Δ is produced. This means that the transitions are shared within this spectral width Δ. Therefore, the density of states needs to take into account the denisty of shared transitions (*D(ε*)Δ). As the interaction between excitons and acoustic-phonons becomes enhanced by increasing temperature, the linewidth Δ(*T*) also depends on temperature. As a result, the stochastic fraction of excitons contributing to recombination within a spectral width Δ(*T*) at *T* can be estimated as


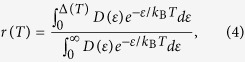


whereby the effective transition strength is given by 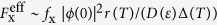
24. As 
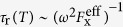
, the essential temperature dependence is determined by *τ*_r_(*T*) ~ Δ(*T*)/*r(T*). As shown in Figs 3(b) and 4(a), a linear increase in the linewidth was observed up to ~25 K due to the exciton-acoustic phonon interaction, leading to a monotonic decrease in the time-integrated PL intensity. For 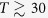 K, the linewidth broadens exponentially as exciton-optical phonon scattering dominates. Also, the PL intensity decreases significantly as other quenching processes are enhanced with increasing temperature. Therefore, the validity of our analysis is limited to temperatures below ~25 K.

As the time-resolved PL intensity in Fig. 4(b) also decays monotonically for 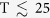 K, the exciton population (*N*) after transient excitation (*g*) can be described by 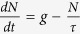, where the PL decay rate (
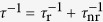
) is a sum of the radiative and non-radiative decay rate. With the population in a steady state *N* = *gτ*, the time-integrated PL intensity at *T* is given by *I(T*) ~ *N(T*)/*τ*_r_(*T*) = *gη(T*), where *η(T*) = *τ*/*τ*_r_ corresponds to the quantum efficiency at *T*^25^. Although *η(T*) is unknown, the ratio of *I(T*) to *I*(5 K) can be used to provide a relative quantum efficiency *ζ(T*), i.e., *I(T*)/*I*(5K) = *η*(T)/*η*(5K) = *ζ*(T). Consequently, the temperature dependence of the radiative decay time can be obtained through the observables *τ(T*), *ζ(T*), and Δ(*T*) as^26^


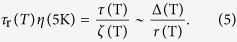


In Fig. 4(c) and (d), the temperature dependence of the radiative decay time *τ*_r_(*T)η*(5K) was obtained from Fig. 4(a) and (b), whereby *r(T*) was also deduced by using Δ(*T*). If the exciton density of states is given by *D(ε*) ~ *ε*^*β*^, we find that Fig. 4(c) and (d) can be fitted well with *β* = −0.38. Usually *D(ε*) was evaluated indirectly through the power factor (*α*) in the temperature-dependent radiative decay time (*τ*_r_ ~ *T*^*α*^), but our analysis provides *β* directly by using the measured *τ*_r_(*T*) and *r(T*). Given that *τ*_r_ ~ *T* and *τ*_r_ ~ *T *^0.5^ were measured in a two-dimensional quantum well (*β* = 0) and a one-dimensional quantum wire (*β* = −0.5), respectively, our result of *β* = −0.38 suggests that our QR can be attributed to the case of a quasi-one-dimensional density of states.

While the exciton emission energy from a QR is determined mainly by the vertical (6~12 nm) and radial confinement (~20 nm), the finely-spaced levels associated with *D(ε*) ~ *ε*^−0.38^ are governed by rotational motion along an orbit in the rim. It is noticeable that the rim orbit length 2*πR* ~ 120 nm for an average QR of radius *R* ~ 20 nm is too small to give a degree of freedom, and the orbital angular momentum becomes quantized. Therefore, the term ‘quasi-one-dimensional’ should be limited to the density of states alone. When the electron and hole are confined in a finite rim, a wavefunction separation between the electron and hole can occur due to various effects such as the effective mass difference, the anharmonic confinement potential along the radial direction, the deformation potential difference in the conduction and valence bands, the strain-induced piezoelectric field, and the local electric field arising from the charge-trapped interface defects^2^,^6^,^7^,^15^,^20^. In this case, a so-called radially polarised electron-hole pair is induced, and the orbits of the electron and hole are different. Additionally, when the Coulomb interaction is considered, the exciton binding energy (~8 meV) of a QR is enhanced compared to that of bulk GaAs (~4.2 meV). As a result, the exciton becomes localised within a finite rim (~20 nm). In this case, the effective trajectory of an excitonic particle within a finite rim could be longer than the average orbit length (2*πR*~120nm), where the integration of a vector potential along the path for the electron and the hole (
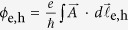
) determines the phase of an excitonic wavefunction, and the phase difference between the electron and the hole (|*ϕ*_e_ − *ϕ*_h_|) gives rise to excitonic AB oscillations.

## Methods

GaAs rings were grown on an n-doped GaAs (001) substrate using a molecular beam epitaxy system with an ion getter pump. After thermal cleaning of the substrate under arsenic ambient at 600 °C, a 100 nm-thick GaAs buffer layar and a 50 nm-thick Al_0.3_Ga_0.7_As layer were grown successively at 580 °C. The substrate temperature was decreased to 310 °C, and Ga metal equivalent to 2 monolayer-thick GaAs was introduced to the substrate at the main chamber pressure of ~3 × 10^−10^ Torr. When the substrate temperature reached 200 °C, arsenic tetramers were introduced to form GaAs rings. Finally, the rings were capped with 60 nm-thick Al_0.3_Ga_0.7_As and 3 nm-thick GaAs for optical measurements^17^,^18^. During ring formation, the flux of Ga is equivalent to a GaAs growth rate of 0.5 monolayer/s, and the flux of arsenic is 1.2 × 10^−7^ Torr. The PL of a single QR was collected at 4 K using a confocal arrangement, where frequency-doubled (400 nm) Ti:sapphire laser pulses (120 fs pulse duration at a 80-MHz repetition rate) were focused on the QR sample (~6 QRs/*μ*m^2^) with a spot-size of 0.8 *μ*m^2^. A time-correlated single photon counting system (TCSPC) was used to obtain the time-resolved PL.

## Additional Information

**How to cite this article**: Kim, H. *et al*. Quasi-one-dimensional density of states in a single quantum ring. *Sci. Rep.* **7**, 40026; doi: 10.1038/srep40026 (2017).

**Publisher's note:** Springer Nature remains neutral with regard to jurisdictional claims in published maps and institutional affiliations.

## Supplementary Material

Supplementary Information
