# Supplementary material for: Quasi-one-dimensional density of states in a single quantum ring
Source: Sci Rep. 2017 Jan 5;7:40026. doi: 10.1038/srep40026 (PMC5213572; doi:10.1038/srep40026)
Supplement: Supplementary Information [file srep40026-s1.pdf]

Supplementary Information for
Quasi-one-dimensional density of states in a single quantum ring

Heedae Kim[1,2], Woojin Lee[1], Seongho Park[1], Kwangseuk Kyhm[1],[*] Koochul Je[3], Robert A. Taylor[2], Gilles Nogues[4], Le Si Dang[4], and Jin Dong Song[5]

[1]*Department of Opto-mechatronics, Cogno-mechatronics, Physics Education, Pusan Nat'l University, Busan 609-735, South Korea*

[2]*Clarendon Laboratory, Department of Physics, University of Oxford, Oxford, OX1 3PU, U.K*

[3]*College of Liberal Arts and Sciences, Anyang University, Gyeonggi-do 430-714, South Korea*

[4]*Department of NANOscience, Institut Néel, CNRS, rue des Martyrs 38054, Grenoble, France and*

[5]*Center for Opto-Electronic Convergence Systems, KIST, Seoul, 136-791, South Korea*

(Dated: November 6, 2016)

[*]Electronic address: kskyhm@pusan.ac.kr

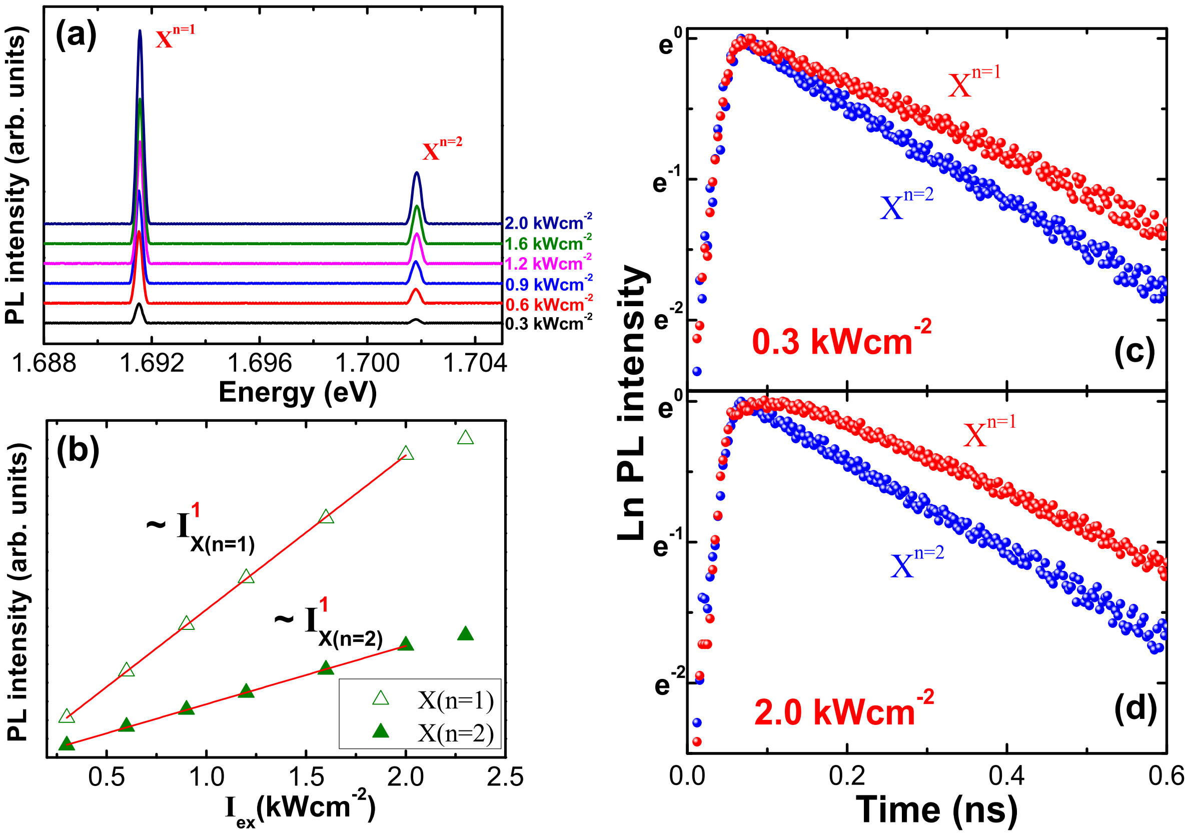

Supplementary Fig. 1: (a) PL spectra of $X^{n=1}$ and $X^{n=2}$ states were measured for increasing excitation intensity, where the both intensities increase linearly up to the saturation excitation intensity **(b)**. Time-resolved PL intensity of $X^{n=1}$ and $X^{n=2}$ at weak ($0.3\,\mathrm{kWcm}^{-2}$) **(c)** and strong ($2.0\,\mathrm{kWcm}^{-2}$) **(d)** excitation intensity.

Supplementary-1: Power dependent and time-resolved PL in a single QR

In addition to the temperature dependence of PL spectrum in Fig. 3(a), we have also performed excitation intensity dependent and time-resolved PL, whereby the low and high energy PL peaks can be attributed to the ground ($X^{n=1}$) and excited ($X^{n=2}$) states, respectively. As shown in Supplementary Fig.1(a), the high-energy PL peak (1.702 eV) appears at $\sim$ 10 meV above the low-energy PL peak (1.692 eV). For increasing excitation intensity, time-integrated PL intensity was plotted in Supplementary Fig.1(b). Both the PL peaks increase linearly up to the saturation excitation intensity although the slope of the low-energy PL ($X^{n=1}$) is three times stiffer than that of the high-energy PL ($X^{n=2}$). Therefore, the high-energy PL results from neither charged exciton nor biexciton state.

At weak excitation intensity ($0.3\,\mathrm{kWcm}^{-2}$), the decay time of the high-energy TR-PL is fast ($\sim 350\,\mathrm{ps}$) compared to that of the low-energy PL ($\sim 570\,\mathrm{ps}$) (Supplementary Fig.1(c)). As excitation is increased, the decay time of the high-energy PL barely changes. However, a transient PL saturation is seen at the low-energy state (Supplementary Fig.1(d)), i.e., the initial decay becomes very slow for $\sim 100\,\mathrm{ps}$. This result suggests the presence of an intra-relaxation from the high-energy state to the low-energy state, whereby the low-energy state becomes saturated. Consequently, we attribute the low- and high-energy PL peaks to the ground ($\mathrm{X}^{n=1}$) and excited ($\mathrm{X}^{n=2}$) states. Provided rotational motion of the exciton is allowed in a quantum ring, fine states are also present between $\mathrm{X}^{n=1}$ and $\mathrm{X}^{n=2}$ states. This can be verified by observing the PL energy oscillation for increasing an external magnetic field, the so-called optical Aharonov-Bohm oscillation because the exciton in a QR changes the different orbital angular momentum state in order to minimize its energy. Regarding our previous report[1], we have estimated that roughly $\sim 10^2$ fine states are possible between between $\mathrm{X}^{n=1}$ and $\mathrm{X}^{n=2}$ states. Therefore, the level spacing ($\sim 0.1\,\mathrm{meV}$) is fine enough to define a density of the states.

Supplementary-2: Theoretical model in a single QR

Supplementary Fig. 2(a) shows one of the AFM images, where the in-plane shape is elliptical and a dip is present in the middle of the QR rather than opening. In order to consider the height-anisotropy, the singly-connected volcano model was utilised[2, 3]. As shown in Supplementary Fig. 2(b), the rim height is maximum along the $[1\bar{1}0]$ direction ($Y = 0$), which is large compared to the rim height along the $[1\bar{1}0]$ direction ($X = 0$). According to the volcano model, the height ($h(\rho,\varphi)$) of a QR-volcano is described in the polar coordinates, where the inner ($h_{\mathrm{inner}}(\rho \leq \widetilde{R}(\varphi))$) and outer ($h_{\mathrm{outer}}(\rho > \widetilde{R}(\varphi))$) structures are given separately as

$$h_{\mathrm{inner}}(\rho,\varphi) = h_0 + \frac{[\widetilde{h}_m(\varphi) - h_0]\{1 - [\rho/\widetilde{R}(\varphi) - 1]^2\}}{\{[\rho - \widetilde{R}(\varphi)]/\widetilde{\gamma}_0(\varphi)\}^2 + 1}, \quad (1)$$

$$h_{\mathrm{outer}}(\rho,\varphi) = h_\infty + \frac{[\widetilde{h}_m(\varphi) - h_\infty]}{\{[\rho - \widetilde{R}(\varphi)]/\widetilde{\gamma}_\infty(\varphi)\}^2 + 1}, \quad (2)$$

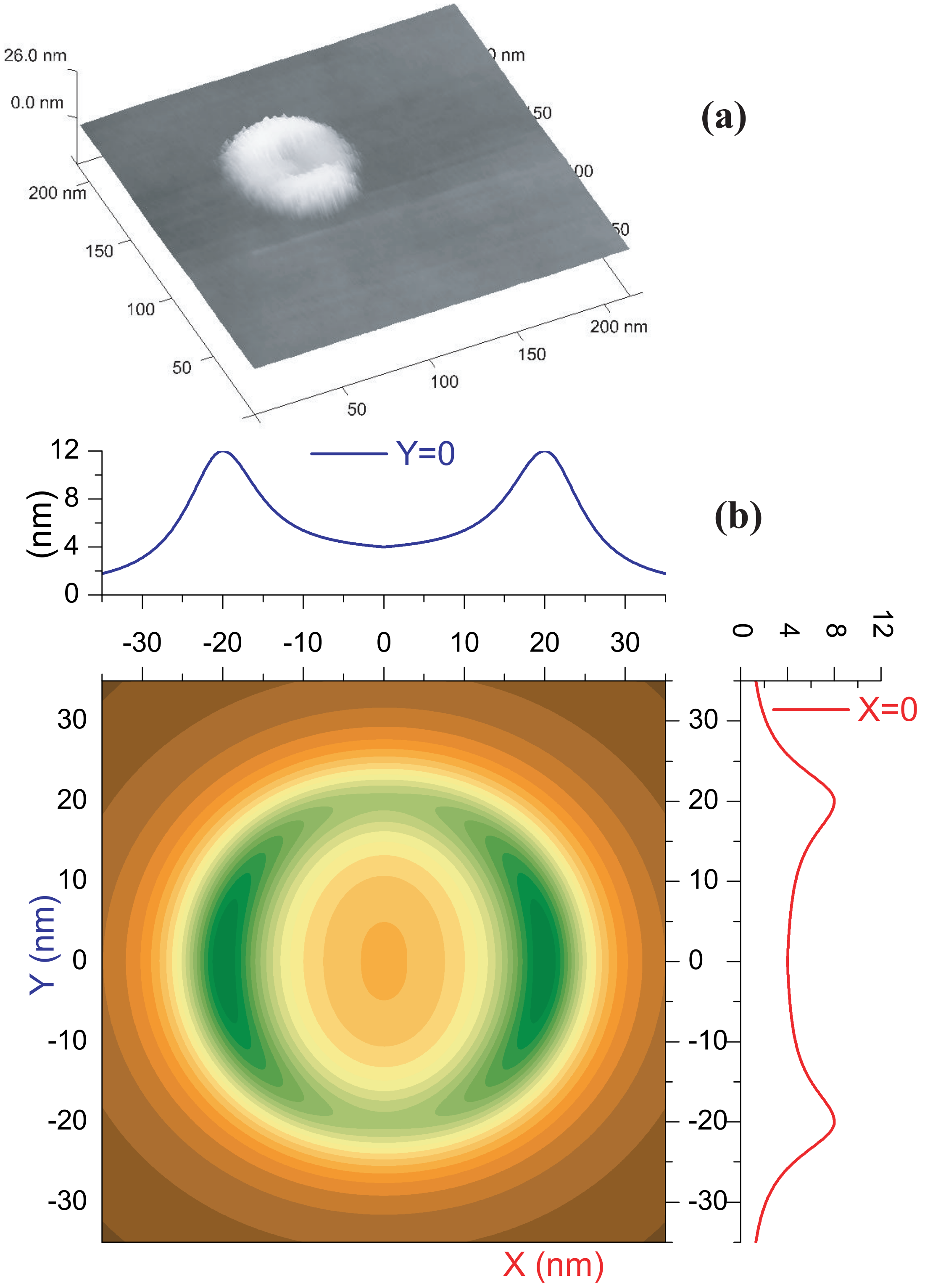

Supplementary Fig. 2: AFM image of a single quantum ring structure **(a)** and volcano-like quantum ring model, where the height anisotropy and the lateral ellipticity are present **(b)**.

where the constant parameters are defined as

$$\begin{aligned}\widetilde{h}_{\rm m}(\varphi) &= h_{\rm m}(1+\xi_{\rm h}{\rm cos}2\varphi), &(3)\\ \widetilde{\gamma}_0(\varphi) &= \gamma_0(1+\xi_\gamma{\rm cos}2\varphi), &(4)\\ \widetilde{\gamma}_\infty(\varphi) &= \gamma_\infty(1+\xi_\gamma{\rm cos}2\varphi), &(5)\\ \widetilde{R}(\varphi) &= R(1+\xi_R{\rm cos}2\varphi). &(6)\end{aligned}$$

While the stiffness of the inner and outer rims are adjusted by the parameters of γ_0 and γ_∞ define, the anisotropy is determined by the parameters of ξ_h, ξ_γ, and ξ_R. h_0, h_m, and h_∞ are also necessary for the height of the volcano center, the rim, and the background off-set, respectively. Regarding a number of atomic force microscope (AFM) images of uncapped GaAs QRs, we obtained the averaged parameters for $R = 20\,{\rm nm}$, $h_{\rm m} = 10\,{\rm nm}$, $h_0 = 4\,{\rm nm}$, $h_\infty = 0.4\,{\rm nm}$, $\gamma_0 = 5.5\,{\rm nm}$, $\gamma_\infty = 5.5\,{\rm nm}$, $\xi_{\rm h} = 0.2$, $\xi_\gamma = 0$, and $\xi_R = 0$.

As the 3-dimensional confinement potential $V(\rho,\varphi,z)$ can be obtained from the anisotropic QR height, the Schrödinger equation is given in the cylindrical coordinates as

$$[-\frac{\hbar^2}{2m^*_{\rm e,h}}\overrightarrow{\nabla}^2_\parallel - \frac{\hbar^2}{2m^*_{\rm e,h}}\frac{\partial^2}{\partial z^2} + V(\rho,\varphi,z)]\Psi^{(e,h)}(\rho,\varphi,z) = E\Psi^{(e,h)}(\rho,\varphi,z). \quad (7)$$

Since the vertical height ($\sim$ 10 nm) is far smaller than the lateral size ($\sim$ 60 nm), the rapidly-varying vertical wavefunction can be separated by the adiabatic approximation [2, 3]. We considered the vertical confinement separately as $V(z,r,\phi) \simeq V(z(r,\phi))$ by using the ansatz

$$\Psi^{(e,h)}(\rho,\varphi,z) = \psi^{(e,h)}_k(z;\rho,\varphi)\phi^{(e,h)}_k(\rho,\varphi), \quad (8)$$

where the vertical quantization along $z-$axis is described by the vertical quantum number k. This gives the Schrödinger equation in the lateral plane as

$$[-\frac{\hbar^2}{2m^*_{\rm e,h}}\overrightarrow{\nabla}^2_\parallel + \varepsilon^{(e,h)}_k(\rho,\varphi))]\phi^{(e,h)}_k(\rho,\varphi) = E^{(e,h)}_\parallel\phi^{(e,h)}_k(\rho,\varphi), \quad (9)$$

where the adiabatic potential $\varepsilon^{(e,h)}_k(\rho,\varphi)$ can be obtained for the corresponding k. Because the vertical confinement energy is included in $\varepsilon^{(e,h)}_k(\rho,\varphi)$, Eq.(9) provides the lateral confinement energy $E^{(e,h)}_\parallel$. We have calculated the adiabatic potential for an electron and a hole separately by using the parameters of a GaAs/AlGaAs structure such as the band offset and

the effective mass in the conduction and valence-band[4]. As no indium component is used in GaAs/AlGaAs structures, the strain effect was ignored.

---

[1] Kim, H. D. *et al.*, Nano Lett. **16**, 27-33 (2016).

[2] Offermans, P. *et al.*, Appl. Phys. Lett. **87**, 131902 (2005).

[3] Fomin, V. M. *et al.*, Phys. Rev. B **76**, 235320 (2007).

[4] Mano, T. *et al.*, Nano Lett. **5(3)**, 425-428 (2005).
